# Cactus cladodes (*Opuntia humifusa*) extract minimizes the effects of UV irradiation on keratinocytes and hairless mice

**DOI:** 10.1080/13880209.2017.1286357

**Published:** 2017-02-09

**Authors:** Kyungmi Park, Hyeon-Son Choi, Yang Hee Hong, Eun Young Jung, Hyung Joo Suh

**Affiliations:** a Regulatory Affairs & Product Compliance Korea and Philippines, Herbalife Korea, Seoul, Republic of Korea;; b Department of Food Science and Technology, Seoul Women’s University, Seoul, Republic of Korea;; c Department of Beauty Art, Suwon Women’s University, Suwon, Republic of Korea;; d Department of Home Economic Education, Jeonju University, Jeonju, Republic of Korea;; e Department of Public Health Sciences, Graduated School, Korea University, Seoul, Republic of Korea

**Keywords:** Hyaluronic acid synthase, hyaluronidase, HA-binding protein, CD44, HaCaT cell, SKH-1 mice

## Abstract

**Context:** Cactus cladodes [*Opuntia humifusa* (Raf.) Raf. (Cactaceae)] is one of the cactus genera, which has long been used as a folk medicine for skin disorders.

**Objective:** This study investigated the skincare potential of cactus cladodes extract (OHE), including its ability to regulate ultraviolet B (UVB)-induced hyaluronic acid (HA) production.

**Materials and methods:** Gene expression levels of hyaluronic acid synthases (HASs) and hyaluronidase (HYAL) were measured in UVB-irradiated HaCaT cells with OHE treatment (10, 25, 50, 100 μg/mL) by real-time polymerase chain reaction (PCR). The HA content was analyzed in hairless mice (SKH-1, male, 6 weeks old) treated with OHE for 10 weeks by using enzyme-linked immunosorbent assay (ELISA). Haematoxylin and eosin (H&E) and immunohistological staining were performed to examine epidermal thickness and levels of CD44 and hyaluronic acid-binding protein (HABP).

**Results:** HA synthases (HAS,1 HAS2, HAS3) mRNA levels were increased by 1.9-, 2.2- and 1.6-fold, respectively, with OHE treatment (100 μg/mL), while UVB-induced increase of hyaluronidase mRNA significantly decreased by 35%. HA content in animal was decreased from 42.9 to 27.1 ng/mL by OHE treatment. HAS mRNA levels were decreased by 39%, but HYAL mRNA was increased by 50% in OHE group. CD44 and HABP levels, which were greatly increased by UVB-irradiation, were reduced by 64 and 60%, respectively. Epidermal thickness, transepidermal water loss (TEWL), and erythema formation was also decreased by 45 (45.7 to 24.2 μm), 48 (48.8 to 25 g/h/m^2^) and 33%, respectively.

**Conclusion:** OHE protects skin from UVB-induced skin degeneration in HaCaT cells and hairless mice.

## Introduction

The primary function of the skin is to serve as an environmental barrier to physical, chemical and biological agents, and protect major internal organs from irradiation. The skin is directly exposed to a variety of environmental hazards, such as chemicals, ionizing and ultraviolet (UV) radiation, and oxygen-derived radicals. Photoaging is mediated by direct UV absorption and reactive oxygen species (ROS)-mediated photochemical reactions. The molecules in the extracellular matrix (ECM) in the skin exhibit specific and characteristic alterations during skin aging. This protective barrier is activated during UV exposure, resulting in direct and indirect damage to cellular and extracellular components, including ECM proteins (Sander et al. 2002). Hyaluronic acid (HA) is a large, ubiquitous glycosaminoglycan found in the ECM and especially abundant in loose connective tissues (Laurent & Fraser 1992; Fraser et al. 1997). It is a high-molecular-weight linear polymer composed of alternating units of d-glucuronic acid and *N*-acetyl-d-glucosamine (Kaya et al. 2005). HA provides structure and viscosity to the ECM because of its polyanionic and hydrophilic properties. HA is synthesized by hyaluronic acid synthases (HASs) and degraded by hyaluronidase (HYAL) on the inner surface of the cell membrane. HAS enzymes add sugar residues and elongate the HASs chain length of synthesized HA. Based on the average chain length, the enzymes are classified as HAS1, HAS2 and HAS3 (Lee & Spicer 2000).

The decrease of HA content has been identified as one of the major factors responsible for the physical appearance of aged skin, including wrinkle formation and loss of skin elasticity (Longas et al. 1987; Ghersetich et al. 1994). Pharmacological agents such as oestrogen (Bentley et al. 1986), retinoic acid (Saavalainen et al. 2005), growth factors and cytokines (Sayo et al. 2002) are known to stimulate HA synthesis and prevent skin atrophy, dryness and wrinkles in aging individuals.

Cactus cladodes [*Opuntia humifusa* (Raf.) Raf. (Cactaceae)] known as Korean Cheonnyuncho, is one of the most valuable and commonly used agricultural commodities in South Korea. It has been used for the treatment of various diseases, such as arteriosclerosis, diabetes mellitus, gastritis and hyperglycaemia (Yoon et al. 2009). *Opuntia humifusa* contains high concentrations of total polyphenols and flavonoids compared to other cactus species. While *O. ficus-indica* (L.) Mill., is better documented in biochemical, biological and pharmacological studies for its anti-inflammatory (Park et al. 2001), anticancer (Chavez-Santoscoy et al. 2009) and antioxidant effects (Butera et al. 2002), *O. humifusa* has a beneficial effect in diabetic rats (Hahm et al. 2011). However, few studies have reported on the effect of *O. humifusa* on photoaging of the skin (Hahm et al. 2011). This study investigated the skincare potential of *O. humifusa* extract (OHE), including its ability to enhance HA production/degradation by UV irradiation in human cell cultures and in hairless mice.

## Materials and methods

### Plant material and preparation of *O. humifusa* extract

Prickly pear cladodes of *O. humifusa* growing in the Jochiwon area (Korea, 363427N 1271744E) were collected in October 2012. The identity of the plant was ascertained morphologically by Prof. K. S. Shin of Kyonggi University, Suwon, Republic of Korea. A voucher specimen (KUCHS-2012008) was deposited at the College of Health Sciences, Korea University, Seoul, Republic of Korea.

The OHE was prepared using a previous method (Kim et al. 2014b) with slight modifications. The cladodes were washed with water, dried with absorbing paper and stored in a refrigerator at 5 °C. Then, 100 g was homogenized in 500 mL of water, and the suspension was hydrolyzed with 5.0 mL of an enzyme mixture (rapidase/viscozyme, 1:3, v/v) under optimum conditions (pH 4.5 and 50 °C) for 6 h. The reaction was stopped by placing the mixture in a boiling water bath for 10 min. The hydrolysate was then centrifuged at 3000 × *g* for 20 min at 5 °C, and the supernatant was stored at −70 °C until further use.

### Cell culture and UVB irradiation

Human immortalized keratinocyte (HaCaT) cells were obtained from Amore Pacific Co. (Yong-in, Korea). HaCaT cells were maintained in Dulbecco’s modified Eagle’s medium (DMEM) (Welgene Inc., Daegu, Korea) supplemented with 10% (v/v) of heat-inactivated foetal bovine serum (FBS) (Welgene Inc.), penicillin G (100 U/mL) (Welgene Inc.) and streptomycin (100 μg/mL) at 37 °C in a humidified atmosphere containing 5% CO_2_ as described previously (Kim et al. 2014c). OHE was treated with HaCaT cells at indicated doses for 1 h, and the cell media were changed with serum-free DMEM, followed by the exposure to UVB using a UV irradiation system (Sankyo Denki Co., Ltd., Tokyo, Japan) with a UV peak at 20 mJ/cm^2^. After the irradiation, more medium was added, and the cells were incubated at 37 °C in a 5% CO_2_ atmosphere for-indicated-periods. To analyze gene expression, cells (1.0 × 10^5^ cells/mL) were cultured in DMEM. After reaching confluence (80%), the cells were irradiated with UVB. Control cells were sham-irradiated.

### Cell viability

Cell viability was quantified using a colorimetric 3-(4,5-dimethylthiazol-2-yl)-2,5-diphenyltetrazolium bromide (MTT) assay, which measures mitochondrial activity in viable cells by a method proposed by Mosmann (1983) with minor modifications. OHE was treated to the cells (24 wells, 2.0 × 10^5^ cells/mL) for 1 h, and thereby cells were exposed to UVB at 20 mJ/cm^2^ by using a UV lamp. UVB-treated cells were incubated for 24 h at 37 °C, after which all wells were aspirated, refilled with MTT (1 mg/mL) solution, and the plate was incubated for 3 h at 37 °C. The formazan crystals formed in actively metabolizing cells were extracted with 10% sodium dodecyl sulphate (SDS) buffer. Absorbance was measured at 540 nm using a microplate spectrophotometer (SpectraMax Gemini, Molecular Devices, Sunnyvale, CA), and the results were expressed as a percentage of the untreated control.

### RNA isolation and real-time polymerase chain reaction

RNA isolation and real-time polymerase chain reaction (real-time–PCR) analysis were performed as previously described (Choi et al. 2015). For measurement of the HAS1, HAS2, HAS3 and HYAL gene expression, total cellular RNA was extracted from HaCaT cells cultured with various concentrations of OHE using the Trizol^®^ reagent (Invitrogen, Carlsbad, CA), according to the manufacturer’s instructions. The total extracted RNA concentration was evaluated by measuring the absorbance at 260 nm, and RNA purity was confirmed as the ratio of absorbance at 260–280 nm (> 1.8). RQ1 RNase-free DNase I (Promega, Madison, WI) was used to remove DNAs in the sample according to the manufacturer's protocol. Then, 1 μg of the total RNA from each sample was reverse transcribed to cDNA using RevertAid™ First Strand cDNA Synthesis Kit (Thermo Scientific Fisher, Waltham, MA). The synthesized cDNA was subjected to real-time polymerase chain reaction (real-time PCR) using a Power Taqman PCR Master Mix kit (Applied Biosystems, Foster City, CA). The data were analyzed using the comparative CT (2^−ΔΔ^*^C^*^T^) method (Livak & Schmittgen 2001). The primers and probes used in *in vitro* analysis included those coding for HAS1 (GenBank ID: NM_008215.2), HAS2 (GenBank ID: NM_008216.3), HAS3 (GenBank ID: NM_008217.4), HYAL (GenBank ID: NM_008317.4) and glyceraldehyde-3-phosphate dehydrogenase (GAPDH; GenBank ID: NM_008084.2) was used as the internal standard. The primers and probes for *in vivo* study are HAS (GenBank ID: NM_008215.2), HYAL (GenBank ID:NM_008317.4) and GAPDH (GenBank ID: NM_001289726.1).

### Hairless mice treatment

The experimental protocol was reviewed and approved by the Korea University Animal Care Committee (KUIACUC-20120926-1). Male SKH-1 hairless mice (6 weeks of age) were purchased from a commercial stock (Central Laboratory Animal Inc., Seoul, Korea). The animals were individually housed in plastic cages with grated stainless steel covers. The colony room was maintained at 24 ± 1 °C with 60% atmospheric humidity and a 12 h light/dark cycle. The animals had access to water and diet *ad libitum* for the entire experimental period.

UVB irradiation was performed according to a previously described method (Sumiyoshi et al. 2010). Briefly, the dorsal skin was exposed to UVB light (FLB20SBL, Sankyo Denki). This source emits light over a wavelength range of 270–400 nm with an output peak at 313 nm. Hairless mice were exposed to UVB irradiation three times per week for 10 weeks in their cages. The initial dose of UVB was set at 36 mJ/cm^2^ and subsequently increased to 54, 72, 108, 122 and 144 mJ/cm^2^ after topical application of the sample. The UV dose was measured using a UV-1700 radiometer (International Light Inc., Shimadzu, Japan).

After an adaptation period, the mice were randomly divided into six groups (six mice/group). OHE was administered to the mice for 10 weeks via two routes: oral administration and/or topical application. The normal control group received non-treated OHE and not exposed to UVB irradiation. The other groups, including the UV-treated control group, were exposed to UVB at a gradient dose. The oral administration groups (OL and OH) were treated with OHE (0.1 and 0.5% in drinking water, respectively). For topical application, OHE Samples were prepared with 15% (v/v) eucalyptol (Sigma, St. Louis, MO) as permeation-enhancer and was applied thrice daily on the dorsal sides of the mice. In the topical application groups (TL and TH), OHE was added to 15% (v/v) eucalyptol (Sigma, St. Louis, MO) to be applied to skin area with 0.2 and 0.4 mg/cm^2^. The consumed volume of drinking water was monitored every 3 days (Table 1).

**Table 1.** Description of the experimental groups.

Groups	UVB irradiation		Oral administration (in drinking water)	Topical application (in 15% ET)
Positive control	Normal	–	–	
Negative control	UVBC	UVB irradiation	water-	15% ET
Oral treatment	OL	UVB irradiation	0.1% OHE	–
	OH	UVB irradiation	0.5% OHE	–
Topical treatment	TL	UVB irradiation	–	0.2 mg/cm^2^ OHE
	TH	UVB irradiation	–	0.4 mg/cm^2^ OHE

OHE: *Opuntia humifusa* extract; ET: eucalyptol. Normal: drinking water without OHE, no UVB irradiation; UVBC: drinking water without OHE plus UVB irradiation; UVB-ET: topical application of vehicle (15% eucalyptol); OL: 0.1% OHE in drinking water plus UVB irradiation; OH: 0.5% OHE in drinking water plus UVB irradiation; TL: topical application of OHE at 0.2 mg/cm^2^ plus UVB irradiation; TH: topical application of OHE at 0.4 mg/cm^2^ plus UVB irradiation.

### Histochemical detection of CD44 and hyaluronic acid-binding protein (HABP) in the skin of hairless mice

All animals were sacrificed, and the dorsal skin was promptly excised. The excised skin (4 × 4 cm) was fixed in 10% neutral buffered formalin to be subsequently processed for histological analysis. Briefly, skin samples were sequentially dehydrated in 70, 95 and 100% ethanol, cleared in xylene, and embedded in paraffin. Serial 4 μm sections were obtained using a microtome and used for histological analyses.

Immunohistochemistry was conducted according to the method proposed by Yamamoto et al. (2008). Immunohistological staining was performed to detect the cell-surface glycoprotein CD44 and HABP. The following antibodies were purchased from commercial sources: anti-CD44 (Abcam, Cambridge, UK), anti-HABP (Thermo, Rockford, IL), and goat anti-rabbit immunoglobulin (Envision Detection kit, Dako, Carpinteria, CA). All the light microscopic and immunohistochemical analyses were performed using a fluorescence stereomicroscope (Leica Microsystems, Wetzlar, Germany). Brown spots represented the CD44 and HABP-2 expression and were evaluated using the Image J program (version 1.28 u, National Institutes of Health, Washington, DC).

### HA content in hairless mouse skin

The HA fraction was prepared from murine skin according to a previously described method (Hashizume et al. 1997) with some modifications. In brief, tissues from the mice were minced and homogenized in 0.1% Triton X-100 in phosphate-buffered saline-calcium and magnesium free (PBS-CMF, pH 7.0) using a Kinematica PT2500E (KINEMATICA, Luzern, Schweiz) at 8000 rpm with cooling condition (∼4 °C). Homogenized samples were centrifuged at 7000 g (BECKMAN COULTER, Seoul, Korea) for 3 h, and the supernatant was collected to be stored at −20 °C. The pellets were suspended in 8 M guanidine-HCl (g/5 mL), followed by the overnight incubation. The extract was centrifuged at above condition, and second supernatant was collected. The supernatants were digested by papain (Sigma-Aldrich, St. Louis, MO).

Hyaluronic acid content was analyzed using the Hyaluronic acid Quantikine^®^ ELISA kit (R&D Systems, Minneapolis, MN). This assay employs a quantitative sandwich enzyme-linked immunosorbent assay (ELISA) technique. Standards, controls and samples were pipetted into wells of a microplate, pre-coated with recombinant human (rh) aggrecan, and any hyaluronic acid present was bound by the immobilized rhAggrecan. After washing away any unbound substances, enzyme-linked rhAggrecan was added to the wells. Any unbound rhAggrecan–enzyme reagent was removed by a wash, then a substrate solution was added to the wells, and a colour developed in proportion to the amount of hyaluronic acid bound in the initial step. The colour development was stopped, and the intensity of the colour was measured at 540 nm.

### Transepidermal water loss and erythema level in hairless mouse skin

Transepidermal water loss (TEWL) was examined using the Tewameter TM 300 (Courage and Khazaka Electronic GmbH) according to the manufacturer’s protocol. The measurement was performed at 40–60% of relative humidity and room temperature (25 °C). A Mexameter MX 18 (Courage and Khazaka Electronic GmbH) was adopted to quantify the degree of erythema levels. Erythema was measured at 660 nm (the spectral absorption peak of haemoglobin) and 568 nm, in order to get rid of the effect of other colour on the reads. The values were expressed as index value (erythema). The accuracy for estimating the erythema contents was ±5%.

### Statistical analyses

All statistical analyses were performed using the Statistical Package for Social Sciences version 12.0 (SPSS Inc., Chicago, IL). Data are reported as the means ± standard deviation (SD) for the HaCaT cell model or the means ± standard error of the mean (SEM) for the hairless mouse model. The significance of the differences was compared using Duncan’s multiple range test. Values of *p* < 0.05 were considered statistically significant.

## Results

### Cytotoxicity of OHE to HaCaT cells

Potential cytotoxicity of OHE was evaluated in cell culture systems. A series of extract concentrations (0–100 μg/mL) was added to the medium, and cells were incubated for 24 h. As shown in Table 2, the cells exhibited a viability of 96.8 ± 6.5% at the OHE concentration of 100 μg/mL without UVB irradiation, suggesting that HaCaT cell does not have significant cytotoxicity within tested ranges of OHE (Table 2). However, HaCaT cells exposed to UVB showed a decreased viability by around 20% compared to the normal group, indicating that UVB has a cytotoxic effect to the cells. Nevertheless, OHE did not show an additive cytotoxicity on UVB-irradiated cells. This result showed that OHE does not give any effect on the viability of cultured HaCaT cells in the presence or absence of UVB irradiation within tested ranges of OHE.

**Table 2.** Relative cell viability of OHE, with or without UVB irradiation (% of normal control).

OHE concentration(μg/mL)	Without UVB irradiation	With UVB irradiation
0	100.0 ± 7.89	81.6 ± 4.2
10	103.2 ± 9.7	83.4 ± 7.2
25	97.3 ± 8.5	82.6 ± 2.8
50	101.3 ± 7.2	80.5 ± 3.8
100	96.8 ± 6.5	80.7 ± 5.2

OHE: *Opuntia humifusa* extract; UVB: ultraviolet B.

### HAS and HYAL gene expression levels

**Figure 1.**
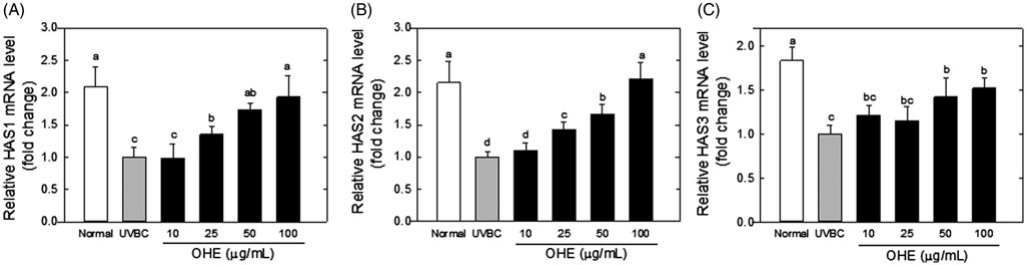
mRNA expression of HAS1, HAS2 and HAS3 in UVB-irradiated HaCaT cells was affected by OHE treatment in a dose-dependent manner. (A) HAS1 mRNA levels, (B) HAS2 mRNA levels, (C) HAS3 mRNA levels. All results expressed a one-fold change compared to the UV-treated control group (UV-Con). HAS: hyaluronic acid synthase; OHE: *O. humifusa* extract; Normal: untreated HaCaT cells; UVBC: UVB-irradiated HaCaT cells (without OHE treatment). The values are the mean ± standard deviation (SD) (*n* = 3). Different letters indicate significant differences (*p* < 0.05) among the treatments as indicated by Duncan’s multiple range test.

Gene expression levels of HAS1, HAS2 and HAS3 were measured in UVB-irradiated HaCaT cells treated with OHE at various concentrations. The results, expressed as fold change relative to UVB irradiation alone (UVBC), are shown in Figure 1. The OHE treatments increased the expression of HAS1, HAS2 and HAS3 in HaCaT cells in a dose-dependent manner compared with the respective levels in the UVB group. HAS1 mRNA expression was increased by around 1.9-fold compared with UVBC group in high dose (100 μg/mL) of OHE treatment, and HAS2 and HAS3 mRNA levels showed 2.2- and 1.6-fold increases, respectively, in 100 μg/mL of OHE treatment. Notably, the expression levels of HAS1 and HAS2 in the UVB-irradiated HaCaT cells treated with OHE at a high concentration (100 μg/mL) did not significantly differ from that in the positive control (normal group).

**Figure 2.**
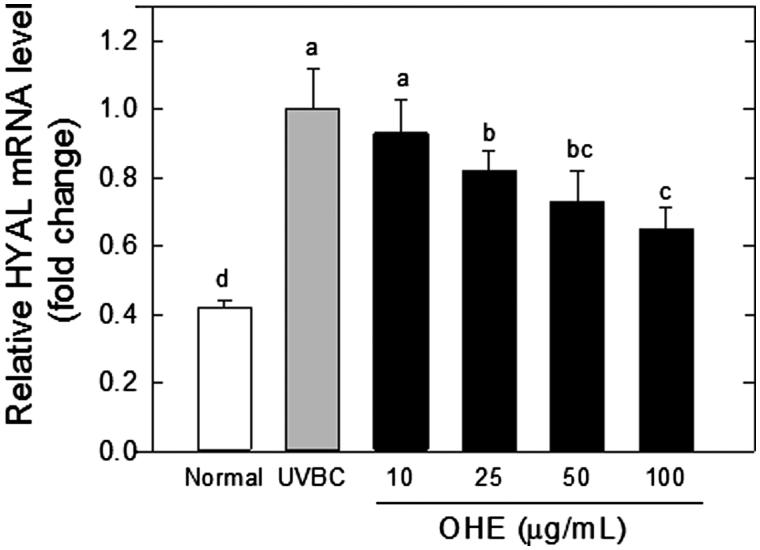
mRNA expression of HYAL in UVB-irradiated HaCaT cells was affected by OHE treatment in a dose-dependent manner. All results expressed a onefold change compared to the UV-Con. HYAL: hyaluronidase; OHE: *O. humifusa* extract; Normal: untreated HaCaT cells; UVBC: UVB-irradiated HaCaT cells (without OHE treatment). The values are the mean ± standard deviation (SD) (*n* = 3). Different letters indicate significant differences (*p* < 0.05) among the treatments as revealed by Duncan’s multiple range test.

Figure 2 presents the effects of the OHE treatments on the HYAL expression in the UVB-irradiated HaCaT cells. The expression levels of HYAL mRNA were significantly (*p* < 0.05) decreased in the presence of OHE in a dose-dependent manner (25 μg/mL, 20%; 50 μg/mL, 28% and 100 μg/mL, 37%) compared to the levels detected in the UVB-treated control (UVBC).

Thus, the OHE treatment significantly increased the HAS expression (HAS1, HAS2 and HAS3), and the OHE-mediated HAS increase was shown to be associated with the down-regulation of HYAL, which degrades HA.

### Epidermal thickness and changes of the CD44 and HABP levels in the skin of hairless mice treated with OHE

**Figure 3.**
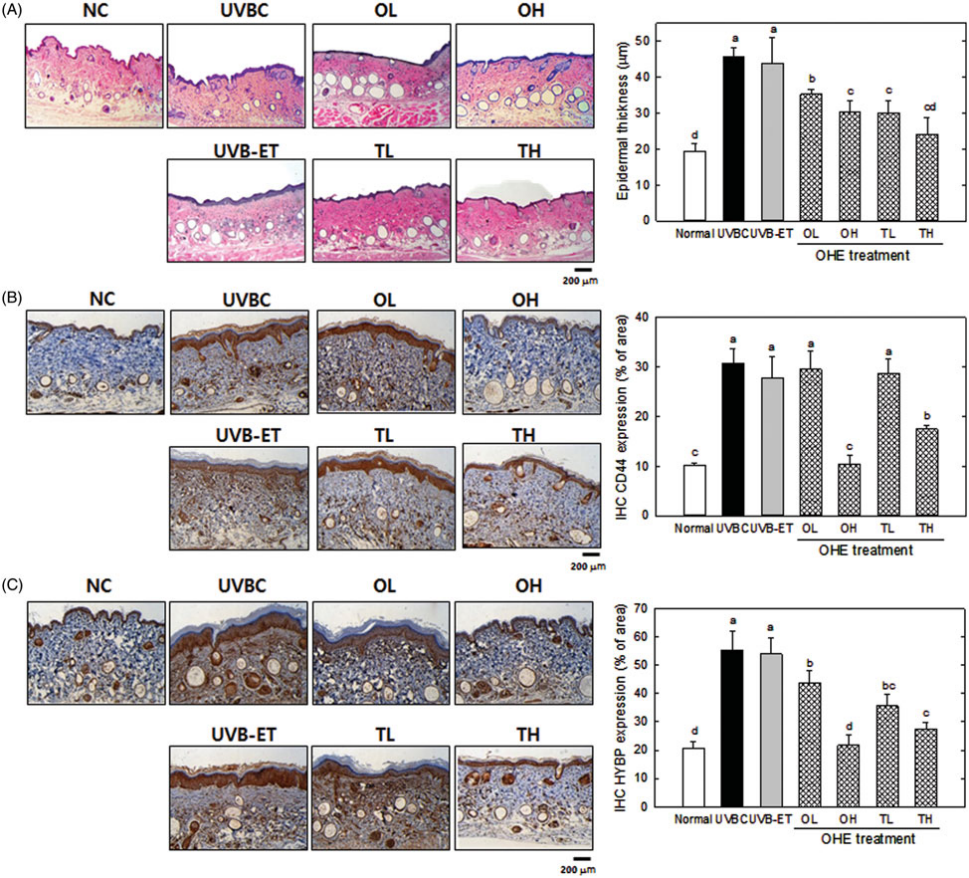
Epidermal thickness, CD44 and HABP expression in the skin of UVB-irradiated hairless mice treated with OHE. (A) H&E staining (epidermal thickness), (B) CD44 distribution and expression, (C) HABP distribution and expression, Normal: drinking water without OHE, no UVB irradiation; UVBC: drinking water without OHE plus UVB irradiation; UVB-ET: topical application of vehicle (15% eucalyptol); OL: 0.1% OHE in drinking water plus UVB irradiation; OH: 0.5% OHE in drinking water plus UVB irradiation; TL: topical application of OHE at 0.2 mg/cm^2^ plus UVB irradiation; TH: topical application of OHE at 0.4 mg/cm^2^ plus UVB irradiation. The values are the mean ± standard error of the mean (SEM) (*n* = 6). Different letters indicate significant differences (*p* < 0.05) among the groups as indicated by Duncan’s multiple range test.

Histological examination was performed using haematoxylin and eosin (H&E)-stained skin specimens. Figure 3(A) shows the effects of UVB irradiation and OHE on epidermal thickness. The exposure to UVB caused a significant increase (*p* < 0.05) in epidermal thickness, which is used as a parameter of skin photoaging. UVB-induced increase of skin thickness was effectively inhibited by OHE treatment. High dose of topical application suppressed the UVB-induced thickness of skin by around 45% compared to the UVBC group (Figure 3(A)).

Immunohistochemical analysis was performed to examine the levels of CD44 and HABP in dorsal skin specimens. The predominant hyaluronic acid receptor on the cell surface of keratinocytes is CD44. The CD44 protein levels were significantly increased (*p* < 0.05) by UVB irradiation (Figure 3(B)). However, the UVB-induced CD44 level was significantly decreased (*p* < 0.05) after oral/topical administration of a high OHE dose (OH) compared with the UVB-treated control (*p* < 0.05). However, low doses of oral and topical treatment did not show significant difference of CD44 level.

Figure 3(C) shows the histochemical results for HABP distribution in the skin of UV-irradiated hairless mice treated by oral administration and topical application of OHE. In this histological analysis, UVB treatment induced the much more brown spots compared with the other groups in the specimen (Figure 3(C)), indicating that HABP expression was increased with UVB exposure. Oral/topical administration of OHE significantly decreased the distribution of brown spots, like the levels of CD44. In particular, high dose of oral administration showed around a 63%-reduction compared to UVBC, with the similar level to the normal control.

### HA content, mRNA expressions of HAS and HYAL in the skin of UV-irradiated hairless mice treated with OHE

**Figure 4.**
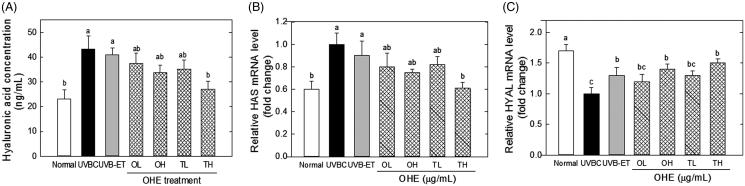
Hyaluronic acid content, and mRNA levels of HAS and HYAL in the skin of UVB-irradiated hairless mice treated with OHE. (A) HA content, (B) HAS mRNA expression and expression, (C) HYAL mRNA expression, Normal: drinking water without OHE, no UVB irradiation; UVBC: drinking water without OHE plus UVB irradiation; UVB-ET: topical application of vehicle (15% eucalyptol); OL: 0.1% OHE in drinking water plus UVB irradiation; OH: 0.5% OHE in drinking water plus UVB irradiation; TL: topical application of OHE at 0.2 mg/cm^2^ plus UVB irradiation; TH: topical application of OHE at 0.4 mg/cm^2^ plus UVB irradiation. The values are the mean ± standard error of the mean (SEM) (*n* = 6). Different letters indicate significant differences (*p* < 0.05) among the groups as indicated by Duncan’s multiple range test.

Figure 4 presents the effects of OHE application on the HA content, and mRNA levels of HAS and HYAL in the skin of hairless mice irradiated with UVB. The HA content in the skin was significantly increased by UVB irradiation, but such an UVB-induced increase of HA content was significantly decreased by OHE topical treatment, even if its difference was not statistically significant OHE oral treatment (Figure 4(A)). High dose of topical treatment reduced HA content by around 37% compared to the UVBC. This result suggests that UVB–induced hyperplasia of the skin includes an increase of HA content with epidermal thickness, and OHE topical treatment inhibits such a UVB-induced hyperplasia. Similarly, HAS mRNA levels significantly increased with UVB irradiation. However, OHE treatment significantly decreased UVB-induced increase of HAS. High dose of topical application showed a 35% decrease compared to the UVBC group (Figure 4(B)). Oral administration and low dose of topical application did not show significant differences. In contrast, HYAL expression levels were decreased with UVB irradiation, and UVB-mediated decrease of HYAL was significantly reversed with OHE treatment. High dose of topical administration increased HYAL expression by around 50% compared to UVBC (Figure 4(C)).

### Transepidermal water loss and erythema level in hairless mouse skin

**Figure 5.**
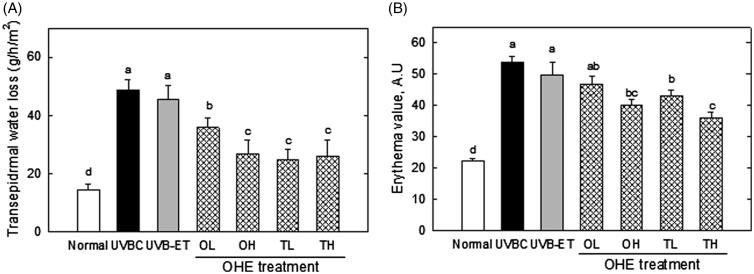
Transepidermal water loss and erythema value in the skin of UVB-irradiated hairless mice treated with OHE. (A) Transepidermal water loss in the dorsal mouse skin was examined using the Tewameter TM 300. (B) The erythema level was determined using a MexameterMX18. Normal: drinking water without OHE, no UVB irradiation; UVBC: drinking water without OHE plus UVB irradiation; UVB-ET: topical application of vehicle (15% eucalyptol); OL: 0.1% OHE in drinking water plus UVB irradiation; OH: 0.5% OHE in drinking water plus UVB irradiation; TL: topical application of OHE at 0.2 mg/cm^2^ plus UVB irradiation; TH: topical application of OHE at 0.4 mg/cm^2^ plus UVB irradiation. The values are the mean ± standard error of the mean (SEM) (*n* = 6). Different letters indicate significant differences (*p* < 0.05) among the groups as indicated by Duncan’s multiple range test.

Figure 5 shows the transepidermal water loss (TEWL) and erythema value in hairless mouse skin. TEWL was examined to evaluate the barrier function of skin in mice irradiated by UV, with or without OHE treatment. UVB-irradiation caused the threefold increase of TEWL compared to the normal group, suggesting that UVB exposure deteriorates skin barrier function by promoting the loss of water from skin (Figure 5(A)). OHE treatment significantly reduced the TEWL value, which increased by UVB treatment (Figure 5(A)), in orally and topically treated groups. High doses of topical OHE treatment decreased TEWL by around twofold compared to the UVBC group (Figure 5(A)). In addition, erythema formation was examined to assess the protective effect of OHE on an undesirable symptom derived from the UVB irradiation. OHE treatment showed an inhibitory effect on UVB-induced erythema formation in mouse skin (Figure 5(B)). In particular, high dose of oral and topical treatment showed a reduction in erythema formation by around 30% (Figure 5(B)). These results indicated that OHE protects the skin from unhealthy states like water loss and erythema formation, which are induced by UVB irradiation.

## Discussion

Natural phenols, which are abundant in plant, have been shown to have photoprotective action when consumed with diet or applied topically (Martorana et al. 2013; Serafini et al. 2014). Several studies demonstrated the protective effects of natural polyphenolic antioxidants (such as flavonoids) against alterations caused by reactive oxygen species (ROS) overproduction (Kim et al. 2014a, 2014b). Our previous studies (Kim et al. 2014b) reported that cactus-originated flavonols, quercitrin and taxifolin, have been changed in their levels via enzyme hydrolysis.

Oi et al. (2012) reported that taxifolin exerted inhibitory effect on the UV-induced skin carcinogenesis, and its action is executed via the interaction of EGFR and PI3K. In addition, another recent study also showed that quercitrin protects the skin by suppressing UVB-induced oxidative stress (Yin et al. 2013). Besides many naturally occurring compounds that have been linked to the UV-induced photodamage in the skin (Nichols & Katiyar 2010; Afaq & Katiyar 2011; Stevanato et al. 2014). Kim et al. (2014a), active flavonoids in *Eruca sativa* Mill. (Brassicaceae), such as quercetin and isorhamnetin, may induce peroxisome proliferator-activated receptor alpha (PPAR-α) activity. *Eruca sativa* and its flavonoid components act as PPAR-α activators and potentially can be used for the development of nutraceutical or therapeutic agents for the treatment of skin barrier dysfunction. Notably, naturally occurring herbal compounds are known to be useful for the prevention of UV-induced adverse effects on the skin (Korać & Khambholja 2011; Rodrigues et al. 2016).

The turnover of HA is rapid, with epidermal HA content turning over within one to two days (Averbeck et al. 2007). Interestingly, it was suggested that UVB irradiation significantly disrupts the turnover of HA in the skin, as well as the expression of HA-metabolizing enzymes, HASs and HYALs (Averbeck et al. 2007; Tzellos et al. 2009).

Our data showed that the expression of HAS1 and HAS2 mRNA in UVB-irradiated cells were restored to the normal level by OHE treatment (Figure 1). A number of studies have reported that HA synthesis can be stimulated by several pharmacological agents (Kuroda et al. 2001; Sayo et al. 2002; Saavalainen et al. 2005). Miyazaki et al. (2003) demonstrated that genistein, daidzein and *Bifidobacterium*-fermented soy milk extract significantly enhanced the production of HA *in vitro* and *in vivo*.

HA catabolism is mediated by the HYAL protein family. Figure 2 shows that the levels of HYAL-1 transcripts in the UV-irradiated HaCaT cells were decreased by OHE treatment. Although HYAL-1 is the major hyaluronidase involved in HA degradation (Kurdykowski et al. 2011), HYAL-1 and HYAL-2 have been described as cooperators in HA degradation. HYAL-2 degrades high-molecular-weight HA to fragments of ca. 50 saccharide units (∼20 kDa), whereas HYAL-1 degrades high-molecular-weight HA or HYAL-2 products of HA degradation to tetrasaccharides (Csoka et al. 2001). Increased HA synthesis and decreased HA catabolism could both lead to elevated HA content in HaCaT cells.

The histological and histochemical changes induced by chronic UV exposure in the skin of hairless mice mimicked those in actinically changed human skin. Exposure of animals to UV irradiation induces glycosaminoglycan alterations in the dorsal skin (Wlaschek et al. 2001). Hyaluronic acid, a high-molecular mass glycosaminoglycan composed of alternating d-glucuronic acid and *N*-acetyl-d-glucosamine residues, is a major component of ECM (Calikoglu et al. 2006). It is well-known that HA retains water, maintains the extracellular space, and facilitates the transport of ionic solutes and nutrients (Tammi et al. 2002). HA is involved in a wide range of cellular functions, including cell proliferation and migration, wound repair, cell locomotion and tumour invasion (Weindl et al. 2004). Takahashi et al. (1996) reported that UVA irradiation significantly increased HA amount in hairless mouse skin. Koshiishi et al. (1999) exposed hairless mice to UV irradiation as solar-simulating irradiation, and found that both hyaluronic acid and dermatan sulphate contents in the dermis significantly increased. These findings suggest that UVB or/and UVA, contribute to the increase in hyaluronic acid and dermatan sulphate contents in the skin.

Margelin et al. (1996) reported that when hairless mice were exposed to UVB irradiation for 10 weeks, glycosaminoglycan content doubled in the skin without any change in the HA/dermatan sulphate ratio. It is generally known that UVB irradiation induces hyperplasia of the epidermis containing HA, thereby indicating that UVB irradiation results in an increase of the HA content in the skin. These studies are correlated to our data (Figures 3 and 4), which showed that epidermal thickness and hyaluronic acid content increased in response to UVB-irradiation in hairless mice experiment (Figures 3(A) and 4). However, these results were contradictory to our *in vitro* data that showed UVB-induced the down-regulation of HAS and up-regulation of HYAL in HaCaT cells (Figures 1 and 2). This discrepancy will be able to be explained in several scenarios. First, excessive UVB exposure is known to be a risk factor to induce damage of the skin, which has complex regulatory systems. Such a regulatory mechanism to maintain homeostasis is shown to work on UVB-induced damage of skin. In other words, HA synthetic mechanisms, which are stimulated by UVB, play some role in minimization of the UVB-induced skin damage by increasing HA content. Therefore, the increase of HA content in UVBC group is shown to be compensatory change of body (or skin) against UVB-induce damage, and OHE treatment is considered to suppress such a compensatory function of UVB. Second, hairless mice skin is expected to show a differential regulation in HA synthesis over dose or time of UVB irradiation. UVB exposure of this study was performed for 10 weeks with maximum 144 mJ/cm^2^. However, this condition seemed to be insufficient to show the UVB-induced decrease of HA content *in vivo*. Dai et al. (2007) showed that chronic UVB irradiation for over 30 weeks with maximum 210 mJ/cm^2^ led to the reduction of HA content with down-regulation of HAS genes. Therefore, the differential effect of OHE according to UVB irradiation period and strength in skin physiology would be examined in the future. Third, this contradictory result of HA content between *in vitro* and *in vivo* may be partially originated from technical error in preparation of the HA fraction; high HA content of dermis in UVBC or UVB-ET group may be mixed to the HA fraction. Therefore, differential analysis of HA on epidermis and dermis would be executed via strict methodological approach in the next study. The effect of UVB irradiation on HA content was different *in vitro* and *in vivo*, but the deleterious action of UVB on the skin was shown to be suppressed by OHE treatment.

The increase of HA content in UVB treatment was shown to be associated with UVB-induced up-regulation of CD44 and HABP in epidermis or dermis (Figure 3(B,C)). High distribution of CD44 and HABP in UVBC was effectively decreased with OHE treatment (Figure 3(B,C)). In particular, the interaction between CD44 and HA is involved in keratinocyte differentiation and lipid synthesis. Tobiishi et al. (2011) reported that their specimens not only showed an elevation in TEWL, which is an indication of barrier disruption, on day 3 after UVB irradiation, but also showed a shift in the average HA molecular mass to a smaller size. Keratinocyte differentiation and lipid synthesis are both induced after barrier disruption (Ajani et al. 2007). Therefore, the interaction between smaller molecular-mass CD44 and HA may be related to barrier recovery after UVB irradiation.

HA has been known to have a space-filling function in the extracellular spaces, maintain tissue hydration and contribute to the regulation of many physiological functions, including cell proliferation, migration, inflammatory responses and wound healing processes, through binding to its receptor, CD44 (Stern & Maibach 2008). However, in our data, UVB-induced increase of HA did not reflect such an enhanced water holding ability; instead, it caused the water loss (Figure 5(A)). This result indicated that HA formed by excessive UVB is not a normal component to perform proper function in skin but a protective by-product against the critical condition. In addition, since HA has been reported to have different physiological functions, depending on its molecular size (Stern & Maibach 2008), it is possible that its water-binding capacity may differ depending on its size.

Our analysis on the transepidermal water loss and erythema formation showed that OHE effectively inhibits water loss and erythema formation, which are induced by UVB irradiation in epidermis. Water holding capacity of epidermis is an important criterion for the evaluation of barrier function of the skin (Gregoriadis 1994), and erythema is an undesirable state of skin caused by various stimuli such as sunburn, and allergies (Sato et al. 2011). Accordingly, OHE was recognized to suppress UVB-mediated degenerations of skin functions.

Cacti fruit and stems have been regarded to be safe for food consumption. They are known to positively affect the body’s redox balance by decreasing oxidative damage to lipids and improve antioxidant status in healthy humans (Zhao et al. 2012). The protective effects of cactus cladode extract against oxidative damage is certainly associated with the presence of several antioxidants such as ascorbic acid, vitamin E, carotenoids, GSH, flavonoids and phenolic acids, which can be detected in fruits and vegetables of different varieties of cactus (Stintzing et al. 2001). Recently, it was found that *O. humifusa*, exerted preventive effects on chemical carcinogenesis in mouse skin, and its preventive actions were associated with reduction of oxidative stress via enhancement of total antioxidant capacity in phase II detoxifying enzyme system (Lee et al. 2012). The other species, *O. ficus-indica* also has been known to have antioxidant activity (Avila-Nava et al. 2014). The consumption of *O. ficus-indica* (300 g) for 3 days significantly increased antioxidant activity in plasma by 20% in subjects.

Current study proved that OHE can significantly stimulate HAS expression to regulate HA synthesis in photo-damaged human keratinocytes. The protective effect of OHE were not due to the growth stimulation of skin cells but because of higher expression of HAS transcripts and lower expression of HYAL transcripts. It was also found that the OHE treatments inhibited the compensatory increases of HA, HABP and CD44 protein expression in hairless mice irradiated with UV, and suppressed the transepidermal water loss and erythema formation. It suggests that OHE protect the skin from the UVB-induced photo damage. Therefore, OHE potentially can be used as an agent for skincare applications.
